# Whole exome sequencing identified sixty-five coding mutations in four neuroblastoma tumors

**DOI:** 10.1038/s41598-017-17162-y

**Published:** 2017-12-19

**Authors:** Aubrey L. Miller, Patrick L. Garcia, Joseph G. Pressey, Elizabeth A. Beierle, David R. Kelly, David K. Crossman, Leona N. Council, Richard Daniel, Raymond G. Watts, Stuart L. Cramer, Karina J. Yoon

**Affiliations:** 1 Department of Pharmacology and Toxicology, University of Alabama at Birmingham, Birmingham, AL USA; 2 Department of Pediatrics, University of Alabama at Birmingham, Birmingham, AL USA; 3 Department of Surgery, University of Alabama at Birmingham, Birmingham, AL USA; 4 Department of Pathology, University of Alabama at Birmingham, Birmingham, AL USA; 5 Department of Pathology and Laboratory Medicine, Children’s of Alabama, Birmingham, AL USA; 6 Department of Genetics, University of Alabama at Birmingham, Birmingham, AL USA; 7 The Birmingham Veterans Administration Medical Center, Birmingham, AL USA; 8 Present Address: Cincinnati Children’s Hospital Medical Center, Cincinnati, OH USA; 9 Present Address: Department of Pediatrics, LSUHSC School of Medicine, New Orleans, LA USA; 10 Present Address: Palmetto Health Children’s Hospital, Columbia, SC USA

## Abstract

Neuroblastoma is a pediatric tumor characterized by histologic heterogeneity, and accounts for ~15% of childhood deaths from cancer. The five-year survival for patients with high-risk stage 4 disease has not improved in two decades. We used whole exome sequencing (WES) to identify mutations present in three independent high-risk stage 4 neuroblastoma tumors (COA/UAB-3, COA/UAB -6 and COA/UAB -8) and a stage 3 tumor (COA/UAB-14). Among the four tumors WES analysis identified forty-three mutations that had not been reported previously, one of which was present in two of the four tumors. WES analysis also corroborated twenty-two mutations that were reported previously. No single mutation occurred in all four tumors or in all stage 4 tumors. Three of the four tumors harbored genes with CADD scores ≥20, indicative of mutations associated with human pathologies. The average depth of coverage ranged from 39.68 to 90.27, with >99% sequences mapping to the genome. In summary, WES identified sixty-five coding mutations including forty-three mutations not reported previously in primary neuroblastoma tumors. The three stage 4 tumors contained mutations in genes encoding protein products that regulate immune function or cell adhesion and tumor cell metastasis.

## Introduction

Neuroblastoma (NB) is an embryonal tumor arising from neural crest cells of the sympathetic nervous system^1^. It is the most common extracranial solid tumor of children, and accounts for ~15% of all childhood cancer deaths. Treatment of children with high-risk disease has been a major challenge in pediatric oncology. Patients less than 18 months of age with low risk disease attain cancer-free status with tumor resection alone or without intervention, due to spontaneous tumor regression^2^. In contrast, patients older than 18 months of age who have high-risk factors such as *MYCN* amplification, bilateral disease, and near-diploid or near-tetraploid karyotype often relapse after initial treatment and remission, with an almost uniformly fatal outcome^3–6^.

The new International Neuroblastoma Risk Group (INRG) Staging System has taken advantage of recent advances in medical imaging and biomolecular diagnostics to establish a consensus for risk stratification^5^. The criteria for classification include stage, age, histology, tumor grade and *MYCN* gene copy number. Criteria for high-risk NB include age greater than 18 months, stage 2 or 3 with *MYCN* amplification, and unfavorable histology^6^.

Genetic abnormalities associated with high-risk stage 4 NB include hemizygous deletions of the q arm of chromosome 11 (up to 62.5% of tumors) and of the p arm of chromosome 1 (25–35% of tumors), and *MYCN* amplification in ~25% of tumors^3,4,7–12^. Gains in the long arm of chromosome 17 (17q21–17qter) is one of the most frequent genetic alterations in NB, occurring 50–70% of all high-risk tumors^3,4^.

Recent advances in next-generation sequencing technology and a collaboration between The Pediatric Tumor Bank and Tumorgraft Development Initiative at Children’s of Alabama and the University of Alabama at Birmingham (COA-UAB) facilitated performing whole exome sequencing (WES) to analyze four recently acquired neuroblastoma specimens. The goals of the study were to sequence the exome of these primary tumors using Whole Exome sequencing to identify mutations, to generate CADD (Combined Annotation Dependent Depletion) scores as a measure of predicted pathogenicity of mutated gene products, and to compare WES data of the stage 3 tumor with the three stage 4 tumors.

## Results

### Clinical characteristics associated with primary neuroblastoma tumors in this study

Primary tumors were received from patients who underwent surgery as standard of care at Children’s of Alabama Hospital (Table 1). Tumors were obtained from patients diagnosed with intermediate (COA/UAB-14) or high-risk disease (COA/UAB-3, COA/UAB-6, COA/UAB-8). Tumors COA/UAB-3 and COA/UAB-6 were *MYCN* amplified. Tumor specimens COA/UAB-3, /UAB-6, and /UAB-8 were obtained from patients older than 18 months, and had high-risk characteristics that included unfavorable histology and *MYCN* amplification.

**Table 1:** Clinical characteristics associated with four primary neuroblastoma tumors.

Tumor ID	Tumor Type	Stage	INRG* Staging	Differentiation (Grade)	MYCN amplified	>18 months
COA/ UAB-3	NB	4	M	Poor	Yes	Yes
COA/ UAB-6	NB	4	M	Poor	Yes	Yes
COA/ UAB-8	NB	4	M	Poor	No	Yes
COA/ UAB-14	NB	3	L2	Poor	No	No

*INRG: International Neuroblastoma Risk Group.

### WES identified 43 mutations not reported previously in four neuroblastoma tumors

WES analysis revealed that each tumor harbored between seven and twenty-five mutations (Table 2). The average of 16 mutations per tumor is consistent with previous reports of 12–18 mutations per tumor^13,14^. The four tumors harbored 43 mutations not previously observed in NB tumors in the dbSNP database (version 138), as well as 22 mutations reported previously to be present in other tumor types^15^. Those 43 mutations are in bold in Tables 2–6. In Tables 3–6, ‘p’ in the third column of each Table identifies the amino acid substitution and position; ‘c’ in this column identifies the nucleotide substitution and position. While no mutation was common to all four tumors, one of the mutations in the *RHPN2* gene was present in two of the four tumors examined: the mutation in this gene (Rhophilin, Rho GTPase Binding Protein 2) was present at nucleotide 217 (G > A encoding Val73Met) in COA/UAB-3 and COA/UAB-8 tumors (Tables 3–5). *RHPN2* contributes to actin cytoskeleton organization, an organelle that regulates cell motility^16,17^. A second mutation introducing a start site of *RHPN2* gene also occurred in tumors COA/UAB-3 and COA/UAB-8. The location of the introduced start site at the intron-exon boundary suggests that this mutation is unlikely to alter the protein product in tumors COA/UAB-3 and COA/UAB-8. A genome-wide association study (GWAS) found that a region containing *RHPN2* has been associated with increased susceptibility to colorectal cancer^18^.

**Table 2:** Summary of variants (mutations) types for all mutations identified in four neuroblastoma tumors.

Variants Types	COA/UAB-3		COA/UAB-6		COA/UAB-8		COA/UAB-14	
	Not reported	Reported	Not reported	Reported	Not reported	Reported	Not reported	Reported
Nonsynonymous coding^1^	**12**	3	**6**	7	**4**	1	**13**	8
Nonsynonymous start^2^								
Splice site acceptor^3^			**1**				**1**	1
Splice site donor^3^								
Start gained^4^	**2**				**1**	1	**1**	
Start lost^4^							**1**	
Stop gained^5^	**2**			1				
Stop lost^5^								
**TOATL # VARIANTS**	**16**	3	**7**	8	**5**	2	**16**	9
**TOTAL # GENES**	**15**	3	**7**	8	**4**	2	**15**	9

^1^Mutation of a single nucleotide, resulting in an amino acid change in the encoded protein; may affect phenotype^66^.

^2^Mutation that occurs in a coding region, at start site.

^3^Mutation that changes nucleotides in genomic loci where splicing takes place.

^4^Mutation that generates a new translation initiation codon in the 5′UTR, or that results in the loss of an initiation codon. Start site loss may result in the loss of protein product.

^5^Mutation that changes the sequence of a codon to create or remove a stop codon (UAA, UAG, UGA).

**Table 3:** WES identified 19 variants in COA/UAB-3 neuroblastoma specimen. Information on each variant (mutation) including gene name, mutation location, mutation type, and known functions/pathways of normal gene product.

Ch#^+^	Gene	Mutation	Mutation type	Known functions/pathways of normal gene product
1*	**TCEB3**	**p.Ala18Val/c.53 C > T**	**Missense**	**Activates RNA polymerase II elongation**
1*	**TOE1**	**p.Ala2Val/c.5 C > T**	**Missense**	**Inhibits cell growth and cell cycle progression**
**1**	**MAEL**	**p.Tyr344Asn/c.1030 T > A**	**Missense**	**Spermatogenesis**
**1**	**SELL**		**Start gained**	**Mediates adhesion**
2*	**WDR35**	**p.Ala1018Asp/c.3053 C > A**	**Missense**	**Promotes CASP3 activation**
2*	**COL4A4**	p.Gly645*/c.1933G > T	**Nonsense**	**Major structural component of basement membrane**
3	MUC4	p.Ala1646Thr/c.4936 G > A	Missense	Plays a role in tumor progression; anti-adhesive properties
**6**	**CLIC5**	**p.Gln50His/c.150 G > T**	**Missense**	**Chloride ion transport**
**6**	**FOXO3**	**p.Glu17Val/c.50 A > T**	**Missense**	**Apoptosis; transcriptional activator**
**13**	**ITM2B**	**p.Ala153Val/c.458 C > T**	**Missense**	**Processing beta-amyloids A4 precursor protein (APP)**
**14**	**RNASE4**	**p.Cys85Phe/c.254 G > T**	**Missense**	**Degrades RNA**
14	ADAM21	p.Pro40Leu/c.119 C > T	Missense	Adhesion protein involved in sperm maturation; epithelial cell function
**17**	**ACADVL**	**p.Phe266Leu/c.798 C > A**	**Missense**	**Mitochondrial fatty acid beta-oxidation**
**19**	**GIPR**	**p.His115Asn/c.343 C > A**	**Missense**	**Pathogenesis of diabetes**
**19**	**RHPN2**	**p.Val73Met/c.217 G > A**	**Missense**	**Binds to and activates GTP-Rho, negatively regulates stress fiber formation and facilitates motility of many cell types including T and B cells.**
19	RHPN2	p.Arg255Gln/c.764 G > A	Missense	Binds to and activates GTP-Rho, negatively regulates stress fiber formation and facilitates motility of many cell types including T and B cells.
**19**	**RHPN2**		**Start gained**	**Binds to and activates GTP-Rho, negatively regulates stress fiber formation and facilitates motility of many cell types including T and B cells.**
19*	**STK11**	p.Arg86*/c.256 C > T	**Nonsense**	**Tumor suppressor**
**20**	**SNX21**	**p.Leu106Pro/c.317 T > C**	**Missense**	**Intracellular trafficking**

+Chromosome number.

*CADD score ≥ 20.

**Table 4:** WES identified 15 variants in COA/UAB-6. Information on each variant (mutation) including gene name, mutation location, mutation type, and known functions/pathways of normal gene product.

Ch#^+^	Gene	Mutation	Mutation type	Known functions/pathways of normal gene product
1	OR2T33	p.Ser87Asn/c.260 G > A	Missense	G-protein receptor activity, olfactory activity
3	C3orf36	p.Pro26Gln/c.77 C > A	Missense	Uncharacterized protein
4*	EVC2	p.Ser270*/c.809 C > A	Nonsense	Hedgehog pathway; bone formation
**6**	**PTPRK**	**p.Ala27Thr/c.79 G > A**	**Missense**	**Cell adhesion and growth, tumor cell invasion and metastasis**
9*	**CDK5RAP2**		**Splice site donor**	**Mitotic spindle orientation, spindle checkpoint activation**
9	PTGES	p.Val37Met/c.109 G > A	Missense	Prostaglandin metabolism
11*	**CRY2**	**p.Gly326Arg/c.976 G > A**	**Missense**	**Circadian rhythm**
**12**	**NPFF**	**p.Gln28Lys/c.82 C > A**	**Missense**	**Modulates morphine-induced effects**
12*	**ATXN2**	**p.Ala1032Thr/c.3094 G > A**	**Missense**	**Negative regulator of EGFR trafficking**
14	ADAM21	p.Pro40Leu/c.119 C > T	Missense	Membrane-bound cell surface adhesion molecule, sperm maturation
14	AHNAK2	p.Leu3217Pro/c.9650 T > C	Missense	Activity may be calcium-dependent
16	POLR3E	p.Ser543Arg/c.1629 C > A	Missense	RNA transcription; DNA-dependent RNA polymerase
**18**	**ARHGAP28**	**p.Lys134Asn/c.402 G > T**	**Missense**	**GTPase activator**
19*	NUP62	p.Asp365Tyr/c.1093 G > T	Missense	Key component of the nuclear pore complex, nucleocytoplasmic transport
**X**	**CYSLTR1**	**p.Leu7Met/c.19 C > A**	**Missense**	**Receptor for cystenyl leukotrienes, bronchoconstriction**

^+^Chromosome number.

*CADD score ≥ 20.

**Table 5:** WES identified 7 variants in COA/UAB-8. Information on each variant (mutation) including gene name, mutation location, mutation type, and known functions/pathways of normal gene product.

Ch#^+^	Gene	Mutation	Mutation type	Known functions/pathways of normal gene product
**2**	**POTEF**	**p.Pro738Ala/c.2212 C > G**	**Missense**	**Involved in retina homeostasis**
**3**	**MUC4**	**p.His1613Asp/c.4837 C > G**	**Missense**	**Tumor progression, cell-cell adhesion, epithelial cell proliferation and differentiation**
6	KIF25	p.Lys28Met/c.83 A > T	Missense	Negative regulator of amino acid starvation-induced autophagy
8	ATAD2		Start gained	Estrogen-induced cell proliferation, cell cycle progression of breast cancer cells
**17**	**KRT31**	**p.Ile37Thr/c.110 T > C**	**Missense**	**Structural component of cytoskeleton, epidermis development**
**19**	**RHPN2**	**p.Val73Met/c.217 G > A**	**Missense**	**Binds to and activates GTP-Rho, negatively regulates stress fiber formation and facilitates motility of many cell types including T and B cells**
**19**	**RHPN2**		**Start gained**	**Binds to and activates GTP-Rho, negatively regulates stress fiber formation and facilitates motility of many cell types including T and B cells**

^+^Chromosome number.

**Table 6:** WES identified 25 variants in COA/UAB-14. Information on each variant (mutation) including gene name, mutation location, mutation type, and known functions/pathways of normal gene product.

Ch#^+^	Gene	Mutation	Mutation Type	Known functions/pathways of normal gene product
1*	**CROCC**	**p.Lys1754Arg/c.5261 A > G**	**Missense**	**Structural component of the ciliary rootlet, a component of centrosome cohesion**
**2**	**RHOQ**	**p.Met80Val/c.238 A > G**	**Missense**	**Epithelial cell polarization**
**2**	**RHOQ**	**p.Met1?/c.1 A > G**	**Missense**	**Epithelial cell polarization**
2	GPAT2	p.Arg621Cys/c.1861C > T	Missense	Regulates glycerolipid biosynthesis
**4**	**CRIPAK**	**p.Cys338Arg/c.1012 T > C**	**Missense**	**Negative regulator of estrogen receptor signaling, regulates cytoskeleton organization**
**6**	**UTRN**	**p.Arg297Gln/c.890 G > A**	**Missense**	**Cytoskeleton/plasma membrane anchoring**
**8**	**DOCK5**	**p.Arg1627Gln/c.4880 G > A**	**Missense**	**Scaffold structure, MAP kinase pathway activation**
**8**	**PSKH2**	**p.Cys3Gly/c.7 T > G**	**Missense**	**Protein serine/threonine kinase activity**
9	RNF20	p.Arg368Trp/c.1102 C > T	Missense	Epigenetic transcriptional activation and gene regulation
**10**	**SUFU**	**p.Ser79Asn/c.236 G > A**	**Missense**	**Negative regulator of hedgehog signaling, negative regulator of beta-catenin signaling**
**11**	**MUC2**	**p.Thr1549Asn/c.4646 C > A**	**Missense**	**Maintain gastrointestinal epithelium, epithelial cell differentiation**
**11**	**KRTAP5–7**	**p.Cys120Tyr/c.359 G > A**	**Missense**	**Keratin intermediate filament protein**
**11**	**CCDC83**		**Splice site acceptor**	
11	KRTAP5–7	p.Tyr98Cys/c.293 A > G	Missense	Hair keratin formation
12	ATF7IP	p.Lys529Arg/c.1586 A > G	Missense	Modulates transcription elongation and histone methylation
**15**	**LYSMD4**	**p.Arg49Trp/c.145 C > T**	**Missense**	**LysM domain containing 4, function not well characterized**
**17**	**AATF**		**Start gained**	**Inhibits histone deacetylase HDAC1**
17	KRTAP4–8	p.Thr173Ser/c.518 C > G	Missense	Keratin-associated protein 4–8
17	KRTAP4–9	p.Asn148Thr/c.443 A > C	Missense	Keratin-associated protein 4–9
17	GRIN2C	p.Val34Met/c.100 G > A	Missense	Involved in excitatory neurotransmission and in neuronal cell death
**19**	**ONECUT3**	**p.Ser313Arg/c.937 A > C**	**Missense**	**Transcriptional activation, cell differentiation, system development**
**19**	**MYO1F**	**p.Arg617Cys/c.1849C > T**	**Missense**	**Actin binding function, cell motility**
**19**	**CYP2A6**	**p.Lys125Met/c.374 A > T**	**Missense**	**Drug metabolism, heme binding; steroid metabolism**
19	LSM14A		Splice site acceptor	Multicellular organism development; regulation of translation
x	AR	p.Gln58Leu/c.173 A > T	Missense	Androgen receptor involved in gene expression, cell proliferation and differentiation

^+^Chromosome number.

*CADD score ≥ 20.

Genes encoding *MUC4* and *ADAM21* also contained mutations in two of the four tumors, but at different loci. Mucin 4 (*MUC4*), a transmembrane mucin expressed predominantly by normal epithelial cells, is involved in cell differentiation, inhibition of cell adhesion, and cell migration^19–21^. MUC4 protein is thought to contribute to tumor metastasis by limiting the adhesion of tumor cells to primary tumor sites. The mutations identified in this gene include the previously reported 4936 G > A encoding Ala1646Thr in COA/UAB-3 and a *not reported* mutation at nucleotide 4837 (C > G encoding His1613Asp) in COA/UAB-8.

The previously reported mutation at nucleotide 119 (C > T encoding Pro40Leu) of the *ADAM21* gene was also present in two of the four tumors (COA/UAB-3 and COA/UAB-6). ADAM21 (A Disintegrin And Metallopeptidase Domain 21) contributes to cell-cell and cell-matrix adhesion and neurogenesis^22,23^.

Each of the three genes (*RHPN2*, *MUC4* or *ADAM21*) that harbored mutations in more than one tumor has a regulatory role in cell adhesion and motility, cell functions essential to the metastatic process^16,19,23–25^.

**Figure 1:**
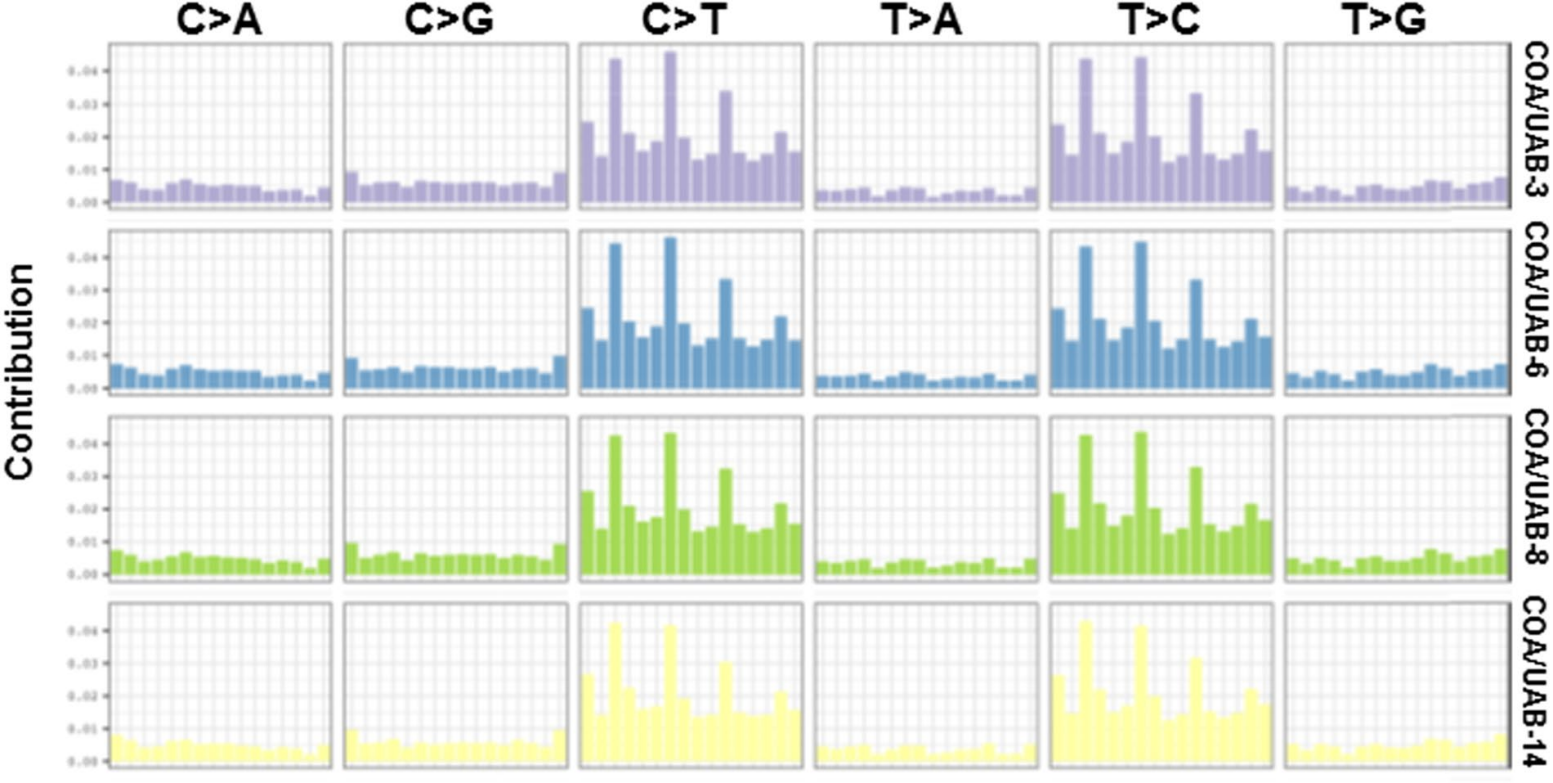
Somatic mutational signature profiling. The somatic motifs for each variant were retrieved from the reference sequence and converted into a matrix. Non-negative Matrix Factorization (NMF) was used to estimate the somatic signature and then plotted. We used SomaticSignatures package to extract the somatic motifs of these samples.

A majority of mutations were nonsynonymous coding mutations, indicating that the genes in which these mutations were present encoded proteins containing amino acid substitutions (Table 2). Additional mutations identified were those that introduced ATG start sites or the splice site acceptor sites at an intron-exon boundary. Among the type of mutations, a majority was found to be missense mutations (Tables 3–6). While some of the mutated proteins contribute to common functions, the wide range of functions affected by mutated genes was diverse as has been seen in previous studies^13,14,26,27^. Further, we retrieved the somatic motifs for each variant from the reference sequence, converted into a matrix to estimate the somatic mutational signature and plotted in Fig. 1. The probability bars (UAB-3: purple, UAB-6: blue, UAB-8: green and UAB-14: yellow) from the 6 substitution subtypes (C > A, C > G, C > T, T > A, T > C, or T > G) are shown in Fig. 1.

### Three of the four tumors harbored genes that had CADD score greater than 20

Tables 3–6 detail genes that harbor mutations, identified by WES. Three of the four tumors had genes with elevated CADD (Combined Annotation Dependent Depletion) scores, a scoring system designed to predict the potential pathogenicity of nucleotide mutations, deletions, or insertions. Scores of ≥20 for a given gene have been associated with specific human pathologies^28^. Genes that were mutated in any of the four tumors analyzed and that had CADD scores ≥20 are described briefly below.

#### COA/UAB-3

Five of the nineteen mutated genes were designated as CADD ≥ 20: *TCEB3* and *TOE1* on chromosome 1, *WDR35* and *COL4A4* on chromosome 2, and *STK11* on chromosome 19. *TOE1* is a target of EGR1 (Early Growth Response 1), and inhibits cell growth^29^. Mutations in the *TOE1* gene have been associated with hepatic and pancreatic malignancies, but the sample number supporting this association is relatively small^15^. Mutations in the *WDR35* gene have been observed in patients with Sensenbrenner syndrome, also known as cranioectodermal dysplasia^30^. Mutations in *COL4A4* have been linked to thin basement membrane disease^31^. Mutations in *STK11* have been associated with Peutz-Jeghers syndrome, a disease characterized by development of hamartomatous polyps in the gastrointestinal tract^32^. Patients with Peutz-Jeghers syndrome have a ~15-fold higher risk of developing intestinal cancer than the normal population^33^.

#### COA/UAB-6

Five of the fifteen mutated genes in this tumor were identified as CADD ≥ 20. These include *EVC2* on chromosome 4, *CDK5RAP2* on chromosome 9, *CRY2* on chromosome 11, *ATXN2* on chromosome 12, and *NUP62* on chromosome 19. *EVC2* (EvC ciliary complex subunit 2) contributes to growth and development of bone and skeleton, and regulates Sonic Hedgehog pathway signaling, a pathway described as essential to NB progression^34–41^. Mutations in the *EVC2* gene have been related to Ellis-van Creveld syndrome and Weyers acrofacial dysostosis^42^. These syndromes are disorders of skeletal dysplasia of the teeth, nails, and bones, respectively^43^.

#### COA/UAB-14

One of the twenty-five mutated genes in this intermediate risk tumor was identified as CADD ≥ 20: *CROCC* on chromosome 1. The protein encoded by the *CROCC* (ciliary rootlet coiled-coil, Rootletin) gene is a major structural component of the ciliary rootlet, and contributes to centrosome cohesion prior to mitosis^44,45^.

### Ingenuity Pathway Analysis (IPA) identified pathways and physiological systems, development and function, and function associated with network using proteins encoded by mutated genes

We next used Ingenuity Pathway Analysis (IPA) to identify pathways (Tables 7–10), physiological systems, and functions likely to be affected by variant proteins encoded by mutated genes (Tables 11–14). P-values indicate the greater or less likelihood that a given protein is involved in a specific pathway. P-values < 0.05 indicate a likely association between indicated proteins and pathways^46^. The range of p-values in Tables 11–14 reflects the likelihood that proteins of interest were related to specific functional subcategories in the broader functional categories indicated in the Table. Functions associated with networks known to include variant proteins encoded by mutated genes are listed in Tables 15–18.

**Table 7:** Pathways identified by IPA to be associated with proteins encoded by mutated genes in COA/UAB-3.

Pathways affected by variant gene products	p-value	Ratio
ERK5 Signaling	0.049	1/63 (0.016)
PXR/RXR Signaling	0.0521	1/67 (0.015)
GPCR Signaling	0.0551	1/71 (0.014)

**Table 8:** Pathways identified by IPA to be associated with proteins encoded by mutated genes in COA/UAB-6.

Pathways affected by variant gene products	p-value	Ratio
Eicosanoid signaling	0.000989	2/63 (0.032)
Prostanoid biosynthesis	0.0067	1/9 (0.111)
Protein kinase A signaling	0.0253	1/386 (0.003)

**Table 9:** Pathways identified by IPA to be associated with proteins encoded by mutated genes in COA/UAB-8.

Pathways affected by variant gene products	p-value	Ratio
RhoA signaling	0.0359	1/122 (0.008)

**Table 10:** Pathways identified by IPA to be associated with proteins encoded by mutated genes in COA/UAB-14.

Pathways affected by variant gene products	p-value	Ratio
CDP-diacylglycerol biosynthesis I	0.0166	1/16 (0.062)
Phosphatidylglycerol biosynthesis II	0.0187	1/18 (0.056)
Sonic hedgehog signaling	0.0309	1/30 (0.0033)

**Table 11:** Physiological systems or functions identified by IPA to be associated with proteins encoded by mutated genes in COA/UAB-3.

Systems affected by variant gene products	p-value (range)	Molecules
Cell-mediated immune response, immune cell trafficking	0.000796–0.0448	SELL, STK11, FOXO3
Embryonic Development	0.000794–0.0482	FOXO3, MAEL, STK11, TOE1, COL4A4, GIPR, SELL, TCEB3
Hematological system development and function, hematopoiesis	0.000796–0.049	SELL, FOXO3, STK11

**Table 12:** Physiological systems or functions identified by IPA to be associated with proteins encoded by mutated genes in COA/UAB-6.

Systems affected by variant gene products	p-value (range)	Molecules
Cell-mediated immune response, immune cell trafficking	0.000149–0.0478	CYSLTR1, PTGES
Hematological system development and function	0.00149–0.00224	CYSLTR1, PTGES, NPFF
Nervous system development and function	0.00149–0.05	CYSLTR1, PTGES, ATXN2, CRY2, NPFF

**Table 13:** Physiological systems or functions identified by IPA to be associated with proteins encoded by mutated genes in COA/UAB-8.

Systems affected by variant gene products	p-value(range)	Molecules
Tissue development	0.000597–0.00119	MUC4

**Table 14:** Physiological systems or functions identified by IPA to be associated with proteins encoded by mutated genes in COA/UAB-14.

Systems affected by variant gene products	p-value	Molecules
Nervous system development and function	0.00105–0.049	UTRN, AR,GRIN2C, RNF20, SUFU
Reproductive system development and function	0.00105–0.048	AR
Skeletal and muscular system development and function	0.00105–0.043	AR, UTRN, SUFU, DOCK5

**Table 15:** Diseases and functions associated with networks identified by IPA to be affected by observed mutations in COA/UAB-3.

ID	Molecules in network	Diseases and Functions associated with this network
1	ACAD10, **ACADVL**, APP, ARMC9, BOD1, C18orf21, C5orf15, C9orf41, C9orf142, CHAC2, CHID1, **CLIC5**, **COL4A4**, DDHD2, GIMAP4, HINT3, **ITM2B**, NENF, NME8, PUS7L, RABL3, **RHPN2**, RNASE11, **SNX21**, TBCC, **TCEB3**, **TOE1**, TOMM5, UBC, UTP23, VWA5A, **WDR35**, WDR88, WDR45B, ZNF720	Cell morphology and organization, neurological disease
2	ADAM18, ADAM20, **ADAM21**, Adam24, ADAM29, ADAM30, Adam26a/Adam26b, ADAMTS6, ADAMTS8, Akt, CNR1, EMR2, ERK, Focal adhesion kinase, **FOXO3**, **GIPR**, GPR55, GPR126, GPRC5C, IFNB1, Insulin, KIRREL3, **MAEL**, Metalloprotease, MMP21, **MUC4**, NMUR2, PI3K (complex), PROKR1, **RNASE4**, **SELL**, SLC22A17, SRC (family), **STK11**, **TAS1R2**	Nervous system development and function, connective tissue disorders, cell-cell signaling

**Table 16:** Diseases and functions associated with networks identified by IPA to be affected by observed mutations in COA/UAB-6.

ID	Molecules in network	Diseases and functions associated with this network
1	**AHNAK2**, ARFGAP2, **ARHGAP28**, **ATXN2**, BMP2, BPHL, C11orf73, C4orf27, CEBPB, **CYSLTR1**, ERK, **EVC2**, FOXP3, GH1, GPR126, GPR137, GPR146, GPR160, GPRC5C, HNF4A, LRRC40, NFkB (complex), NMUR1, NMUR2, **NPFF**, **NUP62**, Orm, **POLR3E**, **PTGES**, **PTPRK**, Srrm1, TMEM176A, UTP3, VN1R1, ZNF71	Carbohydrate metabolism, small molecule biochemistry, tissue morphology
2	APP, ARMC9, BOD1, C18orf21, **C3orf36**, C5orf15, C9orf41, C9orf142, **CDK5RAP2**, CHAC2, CHID1, **CRY2**, DDHD2, EEPD1, GIMAP4, HEATR5A, HINT2, HINT3, LRRC42, MMGT1, NENF, NME8, PUS7L, PYROXD1, RAB43, RABL3, RNASE11, TBCC, TOMM5, UBC, UTP23, VWA5A, WDR88, WDR45B, ZNF720	Developmental disorders, neurological diseases

**Table 17:** Diseases and functions associated with networks identified by IPA to be affected by observed mutations in COA/UAB-8.

ID	Molecules in network	Diseases and functions associated with this network
1	ABCA2, ATAD1, **ATAD2**, ATP6V1F, C1orf123, CDKN1A, CGGBP1, CSNK1G3, DSE, FMR1, HMGXB3, **KIF25**, **KRT31**, MAGEA2/MAGEA2B, MIS18BP1, **MUC4**, MYH8, NPRL2, NSA2, NSF, **POTEE/POTEF**, RAB24, RAB6C/WTH3DI, **RHPN2**, RNASEH2B, SLC30A5, TOR3A, TTLL5, UBC, UQCR11, WDR13, WDR73, ZNF84, ZNF135, ZNF629	DNA replication, recombination and repair, nucleic acid metabolism

**Table 18:** Diseases and functions associated with networks identified by IPA to be affected by observed mutations in COA/UAB-14.

ID	Molecules in Network	Diseases and functions associated with this network
1	**AATF**, **ATF7IP**, C10orf90, CPPED1, **CRIPAK**, **CROCC**, **CYP2A6** (includes others), Cyp2g1, CYP4 × 1, **DOCK5**, ESR1, **GPAT2**, GSTP1, KRT25, KRTAP1–3, **KRTAP4–9**, LOC391322, **LSM14A**, MAPK1, MRI1, **MUC2**, **MYO1F**, NCCRP1, PABPC4L, PCDHB14, RAC1, **RHOQ**, SPRED3, **SUFU**, SUSD1, TMEM150C, UBC, **UTRN**, ZCRB1, ZMAT5	Drug metabolism, small molecule biochemistry, cancer
2	ABCD1, Akr1c19, ALOX15B, AMD1, AMHR2, Androgen-ARA55-AR-ARA70-HSP40-HSP70-HSP90, AQP8, **AR**, AR-HSP90, AR-HSP40-HSP70-HSP90, ATAD2, CHTF18, DISP2, DTX4, ELMO1, **GRIN2C**, HSD17B3, HSP90AA1, KCNG1, MAK, MAPK15, MSMB, MYL3, PATZ1, PI4K2B, PLXNC1, **PSKH2**, RLN1, **RNF20**, Scgb1b27 (includes others), SLC39A8, SMTN, TACC2, TGFB1, ZMIZ1	Organ development, reproductive system development and function

IPA determined that biological functions associated with proteins mutated in the stage 3 COA/UAB-14 tumor included nervous system development and function (p < 0.049), reproductive system development and function (p < 0.048), and musculoskeletal development and function (p < 0.043) (Table 14)- all early developmental processes. In contrast, IPA of genes mutated in the three stage 4 high-risk tumors (Tables 11–13) indicate the potential involvement of cellular functions more closely related to cell-mediated immune response, hematologic development and function, immune cell trafficking, and cell adhesion or motility. Detailed findings by IPA for each tumor are as follows.

#### COA/UAB-3

IPA data indicated that the 19 mutations identified in this tumor were likely to involve proteins that contribute to ERK5 signaling (p = 0.049), PXR/RXR (p = 0.0521), and GPCR signaling (p = 0.0551) (Table 7). ERK5, extracellular-signal-regulated 5, is a member of the MAPK (mitogen-activated protein kinase) family. This pathway is activated by epidermal growth factors which are reported to play key roles in cell proliferation and differentiation^47^. The pregnane X receptor (PXR) is predominantly expressed in the liver and intestine, is usually activated by PXR in conjunction with the retinoid X receptor (RXR), and contributes to drug metabolism by inducing the family of cytochrome P450 enzymes^48,49^. Table 11 shows that the most affected physiological system and development and function in this tumor includes cell-mediated immune response (p < 0.0452), embryonic development (p < 0.0482), hematologic system development and function (p < 0.049), hematopoiesis (p < 0.046), and immune cell trafficking (p < 0.0448).

Nine genes in which mutations occurred in this tumor contribute to cell morphology, cellular assembly and organization, and neurological disease: *ACADVL*, *CLIC5*, *COL4A4*, *ITM2B*, *RHPN2*, *SNX21*, *TCEB3*, *TOE1*, and *WDR35* (Table 15). Nine mutated genes are associated with nervous system development and function, connective tissue disorders, and cell-to-cell signaling or interaction: *ADAM21*, *FOXO3*, *GIPR*, *MAEL*, *MUC4*, *RNASE4*, *SELL*, *STK11*, and *TAS1R2* (Table 15).

#### COA/UAB-6

WES identified fifteen mutations in this tumor (Table 4). IPA analysis demonstrated the likely involvement of the corresponding mutant gene products as components of the following pathways: eicosanoids (p = 0.000989), prostanoid (p = 0.0067), and protein kinase A signaling (p = 0.0253) pathways (Table 8). The eicosanoid pathway is involved in inflammation and immune-related functions, including cyclooxygenase synthesis and metabolism^50^. Prostanoids are the subclass of eicosanoids to which prostaglandins belong. Protein kinase A signaling pathway involves classic endocrine signaling and function to mediate the effect of cAMP^51^. Key physiological systems, functions and development affected by these pathways include cell-mediated immune response (p < 0.00224), hematological system development and function (p < 0.00224), immune cell trafficking (p < 0.0478), and nervous system development and function (p < 0.05) (Table 12).

Ten genes that harbor mutations are involved in carbohydrate metabolism and tissue morphology: *AHNAK2*, *ARHGAP28*, *ATXN2*, *CYSLTR1*, *EVC2*, *NPFF*, *NUP62*, *POLR3E*, *PTGES*, and *PTPRK* (Table 16). Three genes are involved in developmental and neurological disorders: *C3orf36*, *CDK5RAP2*, *CRY2* (Table 16).

#### COA/UAB-8

WES identified seven mutations in this tumor (Table 5). IPA indicated that mutated proteins were associated with Rho A signaling (p = 0.0359), a primary regulator of cell motility (Table 9), including T and B cell motility associated with immune response^52–55^. Tissue development (p < 0.00119) is identified as a key physiological system or function likely to be affected by these mutated gene products (Table 13). Six genes, *ATAD2*, *KIF25*, *KRT31*, *MUC4*, *POTEF*, *RHPN2* in which mutations occurred are involved in DNA replication, recombination and repair, and in nucleic acid metabolism (Table 17).

#### COA/UAB-14

WES identified twenty-five mutations in this tumor (Table 6). IPA predicted that the mutated gene products contributed to CDP-diacylglycerol biosynthesis I (p = 0.0166), phosphatidylglycerol biosynthesis II (p = 0.0187), and sonic hedgehog signaling (p = 0.0309) pathways (Table 10). The sonic hedgehog pathway involvement is consistent with previous reports that this pathway is important for NB cell proliferation and progression^39–41^. Key physiological systems related to mutated gene products include nervous system development and function (p < 0.049), reproductive system development and function (p < 0.048), and skeletal and muscular system development and function (p < 0.043) (Table 14).

Fourteen of the mutated genes in this tumor are involved in metabolism (endogenous molecules as well as drugs), small molecule biochemistry, and cancer: *AATF*, *ATF71P*, *CRIPAK*, *CROCC*, *CYP2A6*, *DOCK5*, *GPAT2*, *KRTAP4–9*, *LSM14A*, *MUC2*, *MYO1F*, *RHOQ*, *SUFU*, and *UTRN* (Table 18). Proteins encoded by four genes are involved in organ morphology, reproductive system development and function (*AR*, *GRIN2C*, *PSKH2*, *RNF20*) (Table 18).

In summary, WES identified a total of sixty-five mutations in one stage 3 and three stage 4 NB tumors. No affected gene or associated cell function was common to all four tumors. The three stage 4 tumors each had mutations in genes encoding aspects of immune function or response. Genes encoding proteins of diverse function were affected, possibly reflecting the phenotypic heterogeneity that has been observed by other methods of analysis for this tumor type.

## Discussion

In our current study, we performed WES analysis of specimens from four primary NB tumors. Three of the four tumors were designated stage 4 and high-risk. Two of the four had amplified *MYCN*. WES identified 43 mutations not reported previously in these tumors. No single mutation was common to all four tumors. Two of *those* mutations and one of the previously reported mutations in *RHPN2*, was identical in tumors COA/UAB-3 and COA/UAB-8 (*RHPN2*, p.Val73Met/c.217 G > A; p.Arg255Gln/c.764 G > A). Each of the stage 4 tumors harbored mutations in genes encoding proteins that directly affect immune function. The mutation frequency in our study was similar to those reported in other studies^13,14,26,27^. *Of note*, we used patient-derived white blood cells as the control to exclude germline mutations which may not contribute to the pathologic process.

*Among successful utilization of WES to identify mutations in NB*, a recent paper by Pugh *et al*. described genetic variations of 240 high-risk NB specimens, and identified genes with significant somatic mutation frequencies (mutation frequencies of < 9.2%) including *ALK*, *PTPN11*, *ATRX*, *MYCN* and *NRAS* which percentages regarded as too low to be identified in a study in which fewer than hundreds of tumors were analyzed^14,26,56,57^. *ALK* has been reported as a major familial NB predisposition gene among high risk NB patients^58^. *ALK* is also a known oncogene in other tumor types such as anaplastic large cell lymphoma^59^. While we observed no *ALK* mutations in our study, this finding is consistent with the low percent of tumors affected (9.2%)^14^. *PTPN11* (mutation frequency of 2.9%), is a member of the protein tyrosine phosphatase family, and alterations of this gene may contribute to NB transformation^14^. We did not detect this mutation in any of 4 NB sequenced. Pugh, *et al*. also identified mutations in the *MYCN* gene. While *MYCN* amplification is a well-documented prognostic indicator for poor outcome in NB, the functional significance of mutations in this gene remains elusive.

Another recent study by Lasorsa *et al*. identified and discussed somatic mutations that may affect cancer progression in NB^26^. WES analysis of 17 high-risk tumors identified 22 mutated genes implicated in cancer progression. In this study, authors also found similar low rates of mutations reported by us and others^13,14,26,27^. Interestingly, Lasorsa *et al*. proposed that *CHD9* and *PTK2* (*FAK*) comprise driver genes associated with aggressive NB, although only 2–4% of tumor specimens examined harbored mutations in these genes. The authors proposed further that loss of *CHD9*, chromatin related mesenchymal modulator, leads to NB tumor progression as seen in other cancer types. The somatic mutations found in *PTK2* localized adjacent to two functional phosphorylation sites (Tyr576 and Tyr861), mutations that activate FAK protein. FAK activation regulates invasive and migratory properties by altering cytoskeletal function and cell adhesion^60–62^. Similarly, we found a mutation in *RHPN2* in two of the three stage 4 tumors. RHPN2 regulates cell invasion and migration by activating RhoA, a master regulator of cell motility^16^. Work is ongoing to evaluate whether *RHPN2* supports NB cell metastasis, and to examine the hypothesis that inhibition of tumor cell motility comprises a therapeutic approach in high-risk NB. Determining functional correlations for the mutations identified is the priority for the next study to strengthen current findings. Further, we acknowledge that our sample number is too small to designate any mutation as a NB driver mutation, which is considered as a limitation of the current study.

In summary, we identified *sixty-five* mutations among four NB tumors using WES, a sequencing method to identify genetic aberrations. Current work focuses on comparing expression profiles and phenotypes of these NB tumors with WES analyses. If genomic characteristics of NB tumors reflect tumor cell phenotype and sensitivity or resistance to specific therapeutic regimens, the observed genomic diversity suggests that personalized approaches to therapy may be necessary to improve clinical outcome for patients with high-risk stage 4 NB.

## Methods

### Ethics Statement: Human Subjects

This study included human subjects. All procedures were approved by the University of Alabama at Birmingham Institutional Review Board (IRB) in accordance with the guiding ethical principles of the IRB respect for persons, beneficence and justice, as embodied in the Belmont Report. Written informed consent and assent were obtained from all participants.

### DNA isolation

Genomic DNA was isolated from primary tumors and white blood cells from each patient using a DNA/RNA extraction kit (EpiCentre, Madison, WI, USA). Purified DNA concentration and quality was determined by ND-1000 spectrophotometer using NanoDrop 3.0.1 (Coleman Technologies, Inc., Wilmington, DE, USA); 260/280 ratios for all DNA preparations ranged from 1.72 to 2.00 (Table S1). DNA samples were submitted for whole exome sequencing by the Heflin Center at UAB (Birmingham, AL, USA). DNA extracted from white blood cells of each patient from whom tumor specimens were received served as controls.

### Whole exome sequencing on Illumina Platforms

Exome capture was performed using the Agilent SureSelect all Human exon v3 capture kit (Agilent SureSelect Human All Exon 50 Mb for target enrichment) by the Heflin Center at the University of Alabama at Birmingham. Briefly, high molecular weight DNA was isolated, and quality checked by electrophoresis using 1% agarose gel to ensure intact high molecular weight DNA. DNA was randomly fragmented using a Covaris S2 sonicator to produce ~200 bp fragments. Fragmented DNA was blunt ended, phosphorylated, and A-tagged to facilitate linker addition. DNA was selected using biotin labeled RNA capture molecules complementary to each exon. Following purification of the exonic sequences by streptavidin-magnetic bead separation, DNA was amplified with primers that introduce a 6-nucleotide index so that samples could be combined in a given lane for sequence analysis. The exonic libraries were run on the HiSeq. 2000 next generation sequencer from Illumina (Illumina, San Diego, CA, USA) with paired end 2 × 100 bp reads.

### WES analysis, depth of coverage

*Whole exome sequencing (WES)* statistics showed that WES performed at the Heflin Center at UAB had a depth of coverage of 39.68 to 90.27, indicating that each base was sequenced a minimum of 39 and a maximum of 90 times. More than 99% of DNA sequences of tumors and corresponding WBCs mapped to specific genomic regions. Over 84% of reads were properly paired, indicating that both forward and reverse reads were correctly oriented. Percent duplication ranged from 9.12% to 34.34%. These parameters indicate the reliability of the WES data presented (Table S2)^63^. Table S3 shows allele fractions of variants not reported previously in each tumor.

### Somatic mutation signature profiling

The SomaticSignatures package was used to extract the somatic motifs of these samples. In brief, the somatic motifs for each variant were retrieved from the reference sequence and converted into a matrix^64^. Non-negative Matrix Factorization (NMF) was used to estimate the somatic signature and then plotted.

### Data analysis and statistics

To call variants (SNPs, INDELs), the raw fastq reads from the exome capture was aligned to ***UCSC’s high19*** reference genome using Burrow-Wheeler Aligner (BWA)^65^. Variants were identified using Broad’s Genome Analysis Toolkit (GATK) and following Broad’s Best Practices for Variant Detection protocol^66,67^. Briefly, the aligned file from BWA was realigned and recalibrated using GATK. Following base recalibration, *MuTect* was used to identify somatic point mutations between the tumor and normal sample^68^. Once the variants were identified, *SnpEff* was then used for annotation^69^.

## Electronic supplementary material

Tables S1 S2 S3
