# Supplementary material for: Whole exome sequencing identified sixty-five coding mutations in four neuroblastoma tumors
Source: Sci Rep. 2017 Dec 19;7:17787. doi: 10.1038/s41598-017-17162-y (PMC5736554; doi:10.1038/s41598-017-17162-y)
Supplement: Supplementary file 1 — Tables S1 S2 S3 [file 41598_2017_17162_MOESM1_ESM.pdf]

Supplementary Information

Whole exome sequencing identified sixty-five coding mutations in four neuroblastoma tumors

Aubrey L. Miller[1], Patrick L. Garcia[1], Joseph G. Pressey[2,8], Elizabeth A. Beierle[3], David R. Kelly[4,5], David K. Crossman[6], Leona N. Council[4,7], Richard Daniel[5], Raymond G. Watts[2,9], Stuart L. Cramer[2,10], and Karina J. Yoon[1*]

[1]Department of Pharmacology and Toxicology, University of Alabama at Birmingham, Birmingham, AL, USA
[2]Department of Pediatrics, University of Alabama at Birmingham, Birmingham, AL, USA
[3]Department of Surgery, University of Alabama at Birmingham, Birmingham, AL, USA
[4]Department of Pathology, University of Alabama at Birmingham, Birmingham, AL, USA
[5]Department of Pathology and Laboratory Medicine, Children's of Alabama, Birmingham, AL, USA
[6]Department of Genetics, University of Alabama at Birmingham, Birmingham, AL, USA
[7]The Birmingham Veterans Administration Medical Center, Birmingham, AL, USA
[8]Current address: Cincinnati Children's Hospital Medical Center, Cincinnati, OH, USA
[9]Current address: Department of Pediatrics, LSUHSC School of Medicine, New Orleans, LA, USA
[10]Current address: Palmetto Health Children's Hospital, Columbia, SC, USA

Table S1. Quantitation of DNA isolated from the primary tumor (T) and matching white blood cell controls (C) indicates that all DNA preparations were suitable for sequencing.

Tumor ID	Concentration: Tumor (T) DNA (ng/µl)	OD[1] 260/280: Tumor DNA	Concentration: Control (C) DNA (ng/µl)	OD[1] 260/280: Control DNA
**COA/UAB-3**	393.7	1.9	384.3	1.72
**COA/UAB-6**	385.7	1.9	252.1	1.85
**COA/UAB-8**	991.4	1.83	303.7	1.86
**COA/UAB-14**	1019.1	1.93	257.9	1.83

[1]Optical density determined by UV spectrophotometer

Table S2. Burrows-Wheeler Aligner (BWA) statistics. BWA statistics summarize % mapped to genome, % properly paired, % duplication and average depth for each tumor specimen.

	Total # raw reads	% mapped to genome	% paired	% duplication	Average depth
**COA/UAB-3 (C)**	102,653,113	102,074,792 (99.44%)	87,902,272 (85.63%)	11.02%	58.33
**COA/UAB-3 (T)**	62,339,903	62,292,642 (99.92%)	61,707,544 (98.99%)	16.62%	39.68
**COA/UAB-6 (C)**	114,039,213	113,319,749 (99.37%)	101,744,579 (89.22%)	9.12%	55.62
**COA/UAB-6 (T)**	78,511,053	78,449,287 (99.92%)	77,541,479 (98.77%)	21.10%	48.07
**COA/UAB-8 (C)**	103,292,357	102,648,729 (99.38%)	87,010,305 (84.24%)	11.96%	58.73
**COA/UAB-8 (T)**	146,122,576	145,834,306 (99.80%)	144,318,249 (98.77%)	29.84%	77.72
**COA/UAB-14 (C)**	90,425,074	90,101,817 (99.64%)	76,685,058 (84.81%)	11.97%	50.82
**COA/UAB-14 (T)**	186,518,027	185,975,508 (99.71%)	184,143,574 (98.73%)	34.34%	90.27

Table S3. Allele fractions of variants (mutations) not reported previously in each tumor. We listed allele fractions (frequency) of variants in four NB samples identified by WES. As shown, >90% of variants were <0.5, supporting the sensitivity of WES in detecting those mutations.

Tumor ID	Allele fraction	# of variants
COA/UAB-3	<0.01	5
	0.1-0.3	6
	0.31-0.5	3
	>0.5	2
	Total variants	16
COA/UAB-6	<0.01	2
	0.1-0.3	0
	0.31-0.5	4
	>0.5	1
	Total variants	7
COA/UAB-8	<0.01	3
	0.1-0.3	1
	0.31-0.5	1
	>0.5	0
	Total variants	5
COA/UAB-14	<0.01	8
	0.1-0.3	7
	0.31-0.5	0
	>0.5	1
	Total variants	16
